# The opinions and thoughts of women who underwent hysterosalpingography for the first time: Letter to the editor

**DOI:** 10.4274/jtgga.2018.0047

**Published:** 2018-08-06

**Authors:** Mehmet Ferdi Kıncı, İlknur Yeşilçınar, Gamze Acavut, Kazım Emre Karaşahin

**Affiliations:** 1 Clinic of Obstetrics and Gynecology, University of Health of Sciences, Gülhane Training and Research Hospital, Ankara, Turkey; 2 Batman University, Health Sciences, Batman, Turkey

To the Editor;

We read the article "The effect of a preprocedural informative video on anxiety levels in patients undergoing hysterosalpingography: A prospective case-control study" by Erkılınç et al. (1) with great interest, which mentioned the effect of a preprocedural informative video on anxiety levels in patients undergoing hysterosalpingography (HSG). They found the Beck Anxiety scores to be significantly lower in the study group compared with the control group. In the literature, there are many intervention studies for pain and anxiety during HSG procedures (2). However, in order to determine the effectiveness of interventions, we believe that the determination of the experiences, opinions, and thoughts of women and information on the procedure before HSG is important (3).

As for our experience, we designed a qualitative study to determine the thoughts and experiences of women towards HSG. This was conducted on 20 women who presented for the first time to have an HSG procedure in Gülhane Training and Research Hospital. Individual interviews were conducted before and after the procedure for each participant.

We used a questionnaire of 14 questions including the sociodemographic and obstetric data of the women. Also, “before and after the procedure” questions were asked to determine the thoughts of the women towards HSG and their answers were recorded using a voice recorder. Before the operation, their sources of information and current feelings were questioned through face-to-face interviews. After the HSG, the interview was recorded again, asking how the overall experience was, and how they would share their experiences with other women, as well as their recommendations to them. The main answers and concerns of women were concentrated around why HSG was done, the pain associated with the procedure, and anxiety caused by the unknown procedure.

After the interviews, we concluded that the knowledge of women was not sufficient on HSG and that they needed to be informed. Women reported that they had gained information about HSG mostly from the internet and from women who had undergone the procedure previously. We determined that the information acquired by women with their own efforts increased their anxiety toward the process. Therefore, it is important to ensure accurate and reliable information provided by health professionals to women who are going to undergo HSG. By doing so, it would be possible to prevent the prejudices, anxiety, and false perceptions/information of women regarding HSG. Therefore, we appreciate the work of Erkılınç et al. (1) because it will have a real positive effect on patients.
