# Supplementary material for: The opinions and thoughts of women who underwent hysterosalpingography for the first time: Letter to the editor
Source: J Turk Ger Gynecol Assoc. 2018 Aug 6;19(3):169–70. doi: 10.4274/jtgga.2018.0047 (PMC6085533; doi:10.4274/jtgga.2018.0047)
Supplement: Related texts

# Reply: Author’s Response

**Authors:** Selçuk Erkılınç, Nazlı Aksoy Kala, Ali İrfan Güzel, Mehmet Çınar, Nafiye Yılmaz

**Affiliations:** 1 Clinic of Gynecologic Oncology, University of Health Sciences, Tepecik Training and Research Hospital, İzmir, Turkey; 2 Clinic of Obstetrics and Gynecology, University of Health Sciences, Ankara Numune Tarining and Research Hospital, Ankara, Turkey; 3 Clinic of Obstetrics and Gynecology, University of Health Sciences, Zekai Tahir Burak Women’s Health Tarining and Research Hospital, Ankara Turkey

Dear Editor,

We thank the authors for their valuable comments on our manuscript that “The effect of preprocedure informative video on anxiety levels in patients undergoing hysterosalpingography: A prospective case-control study”. Hysterosalpingography causes anxiety in patients with infertility and several methods are presented in the literature. Our study showed the benefit of a preprocedural informative video that a simple implementation in clinical practice on reducing anxiety. The authors finding that the main concern of the hysterosalpingography was anxiety caused by the unknown procedure confirmed importance of the management of anxiety during procedure (1). In addition to our study evaluating the patients in a before and after fashion may provide quantity of the change in anxiety levels. So, future studies may be designed in the way that the authors described. The finding of their study that information on procedure mostly obtained from other than health professionals (internet, friends) is interesting. Furthermore, it may cause increase in levels of anxiety. Although a preprocedural informed consent is a standard of care the study concluded that knowledge of patients was insufficient. So, this critic added a perspective to us that a preprocedural informative video may reduce anxiety levels and the main cause of the anxiety was insufficient knowledge of the patients.

## References

[1] Handelzalts JE, Levy S, Peled Y, Binyamin L, Wiznitzer A, Goldzweig G, et al (2016). Information seeking and perceptions of anxiety and pain among women undergoing hysterosalpingography. Eur J Obstet Gynecol Reprod Biol.
